# Application of Chitosan and Its Derivatives Against Plant Viruses

**DOI:** 10.3390/polym16223122

**Published:** 2024-11-07

**Authors:** Tatiana Komarova, Irina Shipounova, Natalia Kalinina, Michael Taliansky

**Affiliations:** 1 Shemyakin-Ovchinnikov Institute of Bioorganic Chemistry, Russian Academy of Sciences, 117997 Moscow, Russia; kalinina@belozersky.msu.ru (N.K.); Michael.Taliansky@hutton.ac.uk (M.T.); 2 Belozersky Institute of Physico-Chemical Biology, Lomonosov Moscow State University, 119991 Moscow, Russia; 3 Vavilov Institute of General Genetics, Russian Academy of Sciences, 119333 Moscow, Russia; 4 National Medical Research Center for Hematology, 125167 Moscow, Russia; 5 The James Hutton Institute, Invergowrie, Dundee DD2 5DA, UK

**Keywords:** chitosan, chitin, plant virus, chitosan nanoparticles, systemic acquired resistance, chitooligosaccharides, plant immunity

## Abstract

Chitosan is a natural biopolymer that is industrially produced from chitin via deacetylation. Due to its unique properties and a plethora of biological activities, chitosan has found application in diverse areas from biomedicine to agriculture and the food sector. Chitosan is regarded as a biosafe, biodegradable, and biocompatible compound that was demonstrated to stimulate plant growth and to induce a general plant defense response, enhancing plant resistance to various pathogens, including bacteria, fungi, nematodes, and viruses. Here, we focus on chitosan application as an antiviral agent for plant protection. We review both the pioneer studies and recent research that report the effect of plant treatment with chitosan and its derivatives on viral infection. Special attention is paid to aspects that affect the biological activity of chitosan: polymer length and, correspondingly, its molecular weight; concentration; deacetylation degree and charge; application protocol; and experimental set-up. Thus, we compare the reported effects of various forms and derivatives of chitosan as well as chitosan-based nanomaterials, focusing on the putative mechanisms underlying chitosan-induced plant resistance to plant viruses.

## 1. Introduction

Climate change and depletion of the natural resources together with human population growth raise the need for an increase in global yield. Numerous plant pathogens cause significant losses of crop yield, forcing farmers to apply potent chemicals that could both accumulate in the environment and be harmful for consumers [1,2]. At the same time, nowadays, there is a focus on sustainable agriculture that includes a reduction in the negative impact of the food production industry to the environment and a search for alternative biosafe and eco-friendly pesticides [3]. Development and implementation of novel approaches to crop plant protection is one of the main directions on the way to productive and sustainable agriculture. There is a demand for innovative and environmentally friendly materials, which could be applied in agriculture for plant protection against various pathogens. Therefore, the biopolymers have widened their range of applications due to their biocompatibility and biodegradability in addition to other beneficial features.

Chitosan (CHT) is a linear polysaccharide composed of β-(1,4)-2-amino-D-glucose and β-(1,4)-2-acetamido-D-glucose units [4]. CHT-based formulations and materials are used in biomedicine, food preservation, agriculture, and other fields (for a review, see, e.g., [5,6,7,8,9]). The source of industrial production of CHT is chitin. Chitin is the second most abundant biopolymer in nature after cellulose. Chitin is a main part of ordered macrofibrils in the exoskeleton of mollusks and crustaceans, as well as in fungi and insect cuticles [10]. Its natural abundance allows obtaining more than 1000 tons every year, of which about 70% comes from marine species [11]. CHT extraction from natural chitin-containing sources (e.g., crab and shrimp shells) requires several steps including demineralization, deproteinization, and deacetylation. Demineralization is performed with strong acids while deproteinization is conducted in alkaline conditions and/or using proteolytic enzymes. Deacetylation is the final step that converts chitin to chitosan. It is performed by treatment with concentrated NaOH or deacetylases; the latter is much less common due to higher costs and lower efficiency [7]. The properties and quality of the resulting CHT preparation highly depend on the conditions (time, concentrations, and temperature) of each step. CHT molecules vary in their molecular weight (MW) depending mainly on the polymerization degree. The polymer length defines its physicochemical and biological properties and, therefore, fields of its practical application [12]. CHT MW is considered one (but not the only) of the main factors defining its solubility in water; there is a reverse correlation between these parameters [13]. There are two main approaches for CHT depolymerization: chemical and enzymatic [14]. Notably, CHT activity could vary depending on the method of its digestion [15]. CHT molecules containing less than 20 units are designated as CHT oligosaccharides. They are highly soluble in aqueous (in the presence of a counterion) and polar solvents. Characterization of CHT chemical structure in addition to the MW includes the deacetylation degree (DD) and the pattern of acetylation [13,16,17]. In contrast to chitin, CHT is a positively charged polymer that is soluble in acidic conditions (pH 5–6) with its $-{\mathrm{NH}}_{2}$ groups protonated to $-{\mathrm{NH}}_{3}^{+}$. The higher the CHT deacetylation degree, the more positive charges are present on its surface [18]. In addition, deacetylated $-{\mathrm{NH}}_{2}$ groups along with –OH groups are available for crosslinking or modification with various compounds that allow for the obtainment of numerous CHT derivatives with novel beneficial properties [6,19]. Taking into account the strong dependency of CHT activity on its MW, one of the main hurdles in CHT application is that the industrially produced batches are often not homogenous and are poorly characterized; their MW is not indicated or is defined in a very wide range (low MW—less than 150 kDa, medium MW—150–700 kDa, and high MW more than 700 kDa) which is not always enough for its further application [12,13]. Usually, MW is determined using viscosimetry, resulting in identification of the average MW but not specifying the range of CHT molecules’ length and the ratio of molecules of each length in the particular batch. On the other hand, such heterogeneous preparations of CHT are likely to be suitable for generation of nanoparticles (NPs) [20]. CHT-based NPs are gaining popularity as a tool for delivery and gradual controlled release of various cargo including pesticides, fertilizers, nutrients, and even nucleic acids [20,21,22]. In addition, they could be regarded as another promising CHT derivative that is believed to possess higher biological activity compared to unmodified CHT.

The beneficial properties of CHT have been known for decades; its activity against plant pathogens was discovered more than forty years ago. The last thirty years have resulted in a burst of studies on CHT properties as a growth stimulator, plant defense inducer, and a perspective biopolymer for production of nanomaterials. CHT was demonstrated to act as an elicitor, i.e., an inductor of the plant defense reactions that activate local and systemic resistance [23,24,25,26]. In addition, CHT could affect some pathogens directly, possessing bactericidal, fungicidal, and nematocidal properties (recently reviewed in [27]). Chitosan was reported to reduce bacterial [28,29] and fungal [30,31,32] growth rate and induce lower fecundity and higher mortality of pests feeding on plants treated with chitosan and its derivatives [33,34,35]. Numerous studies report CHT-mediated induction of plant resistance to viral infection [15,36,37,38,39]. However, the mechanisms underlying CHT antiviral activity are still not clear. Moreover, some results concerning CHT–plant–virus interactions are controversial.

**Figure 1:**
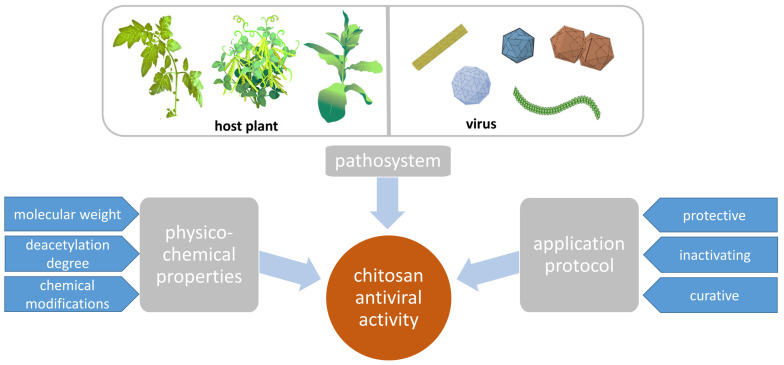
Chitosan activity against plant viruses depends on the plant–virus pair, CHT physico-chemical properties, and the experimental set-up.

Here, we summarize and analyze the data available at present on CHT-induced plant resistance to viral infection, give an overview of the tested pathosystems with a particular focus on the experimental set-up and protocol of CHT application, and make an effort to compare the effect of various CHT preparations with regard to their homogeneity, MW, DD, and chemical modifications (Figure 1). In addition, we pay special attention to studies of CHT-based NPs’ antiviral effect. And, finally, we discuss the putative mechanisms of CHT activity against plant viruses.

## 2. Antiviral Activity of CHT

### 2.1. Pathosystems

CHT possesses antiviral properties as was demonstrated in various pathosystems. Plants of different families, mainly from *Fabaceae* and *Solanaceae*, along with some exotic plants such as papaya, passiflora, and others were used as hosts for diverse viruses from nine families as well as viroids in experiments where CHT was tested as an antiviral agent (Table 1). Members of the *Fabaceae* family appeared to be the most sensitive to CHT: foliar application of CHT solution induced a high degree of resistance (up to 100%) to different virus infections (tobacco necrosis virus, alfalfa mosaic virus, bean golden mosaic virus, peanut stunt virus, and bean mild mosaic virus) in bean and pea plants [36,37,38,39,40,41]. Notably, the effect did not depend on the type of response to the virus—systemic infection or local lesions; in both modes of infection, the reduction in virus accumulation or plant resistance was observed. Numerous experiments on *Solanaceae* plants—*Nicotiana tabacum*, *N. benthamiana*, *Solanum lycopersicum*, *S. tuberosum*, and *Datura stramonium*—showed that depending on the virus, the host plant, and the experimental set-up, the effect of CHT application was different. For necrotic hosts, the reduction of local lesions number varied from 90% to 20% [15,36,40,42,43,44,45,46,47]; for other host plants, milder symptoms or even no symptoms were observed, and the number of plants with successful systemic infection was reduced by 30–60% [40,46,47] (Table 1). Of note, CHT treatment did not launch any resistance to the viral infection in *Brassica campestris* ssp. *rapa* cv. Just Right, a member of the *Brassicaceae* family, as was shown for cauliflower mosaic virus, turnip mosaic virus, and radish mosaic virus [40]. Taking into account that all three viruses are representatives of three different families, it is likely that the absence of the effect is plant dependent. The study by Jia et al. [48] supports this assumption: another member of the *Brassicaceae* family, *Arabidopsis thaliana*, was treated with a 0.005% solution of CHT oligosaccharides and 1 day later challenged with tobacco mosaic virus (TMV). It was demonstrated that TMV coat protein content was 4-fold reduced in these plants compared to the control infected group. In addition, pre-treatment with CHT resulted in a ~25–30% decrease in disease index and the amount of necrotic cells in the TMV-inoculated leaves [48]. Moreover, it was shown that CHT-induced TMV resistance is associated with activation of a salicylic acid-mediated defense pathway. Of note, it is a member of the *Brassicaceae* family, *Brassica campestris* L. ssp. chinensis (L.) Makino, from which a chitosan-binding protein was isolated [49], and it could not be excluded that it serves as a putative chitosan receptor.

Thus, the members of the *Brassicaceae* family are also sensitive to CHT treatment which launches multiple defense-associated pathways [50]; however, more studies are needed to elucidate the mechanism of the CHT-induced response of these plants to viral infection.

Interestingly, to our knowledge, there are no studies on CHT-induced antiviral resistance in monocot plants, despite numerous papers that have been published on the activation of the resistance to other pathogens and to abiotic stresses (reviewed in [51,52]).

To summarize, CHT-induced antiviral resistance has been demonstrated in most of the tested plant–virus combinations independently of taxonomic groups. It is likely that members of the *Fabaceae* family are more responsive to CHT treatment, but it should be taken into account that only two species were tested—*Phaseolus vulgaris* and *Pisum sativum*—while at least seven species (and their cultivars) representing the *Solanaceae* family were used as hosts for various viruses in experiments where CHT antiviral activity was assessed. In addition, CHT ability to induce antiviral resistance was demonstrated in members of other families: passiflora, papaya, quinoa, and cucumber.

**Table 1:** Chitosan application for induction of plant resistance to viral infections.

Plant	Virus	Type of Chitosan	Treatment Protocol	Effect	References
*Phaseolus vulgaris* (bean)	Tobacco necrosis virus (TNV)	CHT from the Antarctic krill; CHT with a 76 kDa MW and a 85% deacetylation degree (DD)	Plant treatment with 0.1 or 0.15% CHT solution 1 day before inoculation	Reduction in the number of TNV-induced local lesions by 75–100%	[36,39]
	Alfalfa mosaic virus (AMV)	CHT from krill and crab	(1) 0.1 to 0.00001% CHT 1 day before inoculation (2) Short-time (applied for 5 min and rinsed with water) treatment with 0.01–0.25% CHT 1 day before inoculation (3) 0.1 to 0.00001% CHT mixed with inoculum (4) The lower leaf sprayed with 0.1% CHT solution and non-treated leaf of the same plant was inoculated	The highest efficiency of inhibition was shown for 0.1–0.001% CHT solutions; however, when applied together with inoculum, CHT solution even in 0.00001% concentration inhibited viral infection. Moreover, 40–60% of local lesion reduction was observed in non-treated leaves of the CHT-sprayed plants.	[36,37]
	Bean golden mosaic virus (BGMV)	NS	Plant treatment 1 or 3 times weekly with 0.1% CHT solution before inoculation	Protection from infection: no symptoms on CHT-treated plants were detected 3 weeks after inoculation	[40]
	Peanut stunt virus (PSV)	CHT from the Antarctic krill	Plant treatment with 0.1% CHT solution 1 day before inoculation	Reduction in the number of systemically infected plants by 50–75%	[36]
	Bean mild mosaic virus (BMMV)	Four chitosan fractions with an MW of 1.2, 2.2, 10.1, and 30.3 kDa as well as non-fractionated CHT with an average MW 40.4	Plant treatment (spray) with 0.001 or 0.01% CHT solution before inoculation	All tested fractions as well as unfractionated CHT suppressed infection development in the inoculated leaves for at least 8 days. Plants treated with the low-molecular -weight CHT (Mw = 2.2 and 1.2 kDa) displayed no systemic infection by the 14th day after inoculation	[38]
*Pisum sativum* (pea)	AMV, PSV	CHT from the Antarctic krill	Plant treatment with 0.1% CHT solution 1 day before inoculation	Reduction in the number of systemically infected plants by 50–75%	[36]
*Solanum lycopersicum* (tomato)	Potato spindle tuber viroid (PSTV)	CHT from the Antarctic krill	(1) 0.001–0.1% CHT solution was added to inoculum 10 min before plant treatment (2) 0.1% CHT solution was applied as a foliar spray 1 day before inoculation (3) Plants were treated with 0.1% CHT solution 1, 3, 5, 7, or 24 h after inoculation	Inactivating and protective treatment gave the best effect: 85–100% of plants were not infected after 0.01 or 0.1% CHT application; protective treatment resulted in an average of 78% resistant plants. Curative treatment was effective only for first 3 h (60–80% of resistant plants)	[53]
	Tobacco mosaic virus (TMV), potato virus X (PVX)	CHT from the Antarctic krill	Plant treatment with 0.1% CHT solution 1 day before inoculation	Reduction in the number of systemically infected with PVX plants by 50–75%	[36]
	Cucumber mosaic virus (CMV)	CHT with an MW 50–190 kDa and a 75–85% DD	Plants were sprayed with 0.1% CHT solution (10 mL per plant) and inoculated with CMV 24 h later	Significant reduction in CMV accumulation in plants treated with CHT at the 20th (up to 86%) and the 90th (100% virus elimination) day after inoculation	[46]
	Tomato leaf curl virus (ToLCV)	NS	Tomato seeds were soaked in 5% CHT solution, and 25 days after sowing, leaves were sprayed with 0.1% CHT	Reduction in disease severity by ~85% and 75% on the 45th and 75th day after inoculation	[54]
*Solanum tuberosum* (potato)	PVX, PVS, AMV	CHT with an MW of 3 and36 kDa and 85% DD was obtained from crab CHT using enzymatic digestion; 120 kDa CHT with 69% DD was obtained from krill	Non-infected potato plants were sprayed with CHT solution (0.1%), and 1, 2, 3, or 4 days later, leaves were (1) detached and inoculated with PVX; after that, 1 cm disks were cut off from these leaves and incubated for 6 days in Petri dishes on the surface of distilled water (2) The treated leaves (still attached to the plant) were inoculated with PVX, and the efficiency of systemic infection was assessed 3 weeks later in the upper non-inoculated leaves (3) Cuttings from plants infected with PVX or PVS were put into the liquid Murashige and Skoog medium with or without CHT; virus accumulation levels were assessed after a month (curative treatment)	(1) The disks from treated leaves (120 kDa CHT had the best effect) accumulated a significantly lowerconcentration of the virus than the control (2) The whole plants sprayed with CHT and infected with PVX demonstrated resistance to PVX (120 kDa CHT treatment gave the best effect) (3) No reduction in PVS or PVX levels in the infected plants was observed (no curative effect)	[55]
*Nicotiana tabacum* (tobacco)	TMV	CHT from the Antarctic krill; CHT preparations of high MW (130–500 kDa) or low MW (from 2 to 17.0 kDa) and different DD	Plant treatment with 0.1% CHT solution 1 day before inoculation; inactivating treatment (CHT mixed with inoculum); protoplasts incubation with 0.1 or 0.01% CHT	Reduction in the number of TMV-induced local lesions by ~20–50% depending on experimental set-up. Low-MW CHT (2–17 kDa) was shown to be the most effective (up to 90% reduction in the number of TMV-induced local lesions). Tobacco protoplasts’ incubation with CHT led to their partial resistance to TMV	[15,36,42,44,45]
	TNV	CHT with a 85% DD and a 2500–3000 polymerization degree (MW ~ 400–500 kDa)	Plant treatment with 0.1% CHT solution 2 days before inoculation	Reduction in the number of TNV local lesions by a range from 32% to 83%; BY-2 cells incubated with CHT (from 0.01 to 0.1%) demonstrated typical morphological features of apoptosis	[43]
	CMV	CHT with an MW of 50–190 kDa and a DD of 75–85%	Plants were sprayed with 0.1% CHT solution and inoculated with CMV 24 h later	11 days after inoculation, CHT-treated plants showed no symptoms while untreated plants showed mosaic	[46]
*Nicotiana glutinosa*	CMV, pepper mild mottle virus (PMMoV)	600 kDa CHT, 80–95% DD, and a compound obtained from CHT and ammonium polyphosphate (P-CHT)	Foliar application of 0.01%, 0.05%, and 0.1% solution of CHT or P-CHT daily 3 times before inoculation	The number of PMMoV local lesions reduced depending on CHT concentration: the best effect was obtained for plants treated with 0.1% CHT (~75% decrease), while P-CHT appeared to be less effective	[47]
*Capsicum annum* (chili pepper)	CMV	600 kDa CHT, 80–95% DD, and a compound obtained from CHT and ammonium polyphosphate (P-CHT)	Foliar application of 0.01%, 0.05%, and 0.1% solution of CHT or P-CHT daily 3 times before inoculation	The accumulation of CMV (as detected via ELISA) in the plants treated with 0.1% CHT- or P-CHT was lower by ~30% compared to the untreated plants; moreover, the symptoms of infection in the treated plants were markedly less severe	[47]
*Datura stramonium* (stramony)	TMV, figwort mosaic virus (FMV)	NS	Plant treatment with 0.2% CHT solution weekly 3 times before inoculation	The percent of plants with FMV symptoms decreased 3-fold compared to untreated plants. Local lesion production caused by TMV was moderately inhibited on CHT-treated young leaves	[40]
*Arabidopsis thaliana*	TMV	CHT oligosaccharides	Plants were treated with 0.005% CHT oligosaccharides and 1 day later challenged with TMV	TMV coat protein content was 4-fold reduced in CHT-treated plants compared to the control infected group. In addition, pre-treatment with CHT resulted in a ~25–30% decrease in the disease index and amount of necrotic cells in the TMV-inoculated leaves	[48]
*Brassica campestris* (turnip) *	Cauliflower mosaic virus (CaMV)	NS	Plant treatment with 0.1 or 0.3% CHT or its chemically modified polyanionic form solution a day before inoculation	2–3 weeks after infection, no protective effect of CHT was observed (symptoms developed in the same manner—time and severity—as for non-treated plants); virus accumulation was confirmed via ELISA	[40]
	Turnip mosaic virus (TuMV), radish mosaic virus (RaMV)	NS	Plant treatment with 0.1 or 0.3% CHT	2–3 weeks after infection, no protective effect of CHT was observed (symptoms developed in the same manner—time and severity—as for non-treated plants); virus accumulation was confirmed via ELISA for TuMV or double immunodiffusion test in agar gel for RMV	[40]
*Cucumis sativus* (cucumber)	Squash mosaic virus (SqMV)	NS	Seeds treatment with 0.9% CHT solution for 1 h. 2, 4, and 6 weeks after planting, leaves were sprayed with the same solution	CHT application significantly delayed appearance of symptoms and reduced disease severity as well as virus titer, especially in the generative phase	[56]
*Carica papaya* (papaya)	Papaya ringspot virus (PRSV)	NS	Root irrigation andfoliar spraying of papaya plants with 200 µL of 0.5% CHT solution 1 day before virus inoculation; treatment was performed at seedling stage or at fruiting stage	The disease index (based on evaluation of symptoms severity) was reduced in CHT-treated plans more than two-fold compared to control untreated plants; the difference was registered up to the 42nd day after inoculation	[57]
*Passiflora spp*. (passiflora)	CMV	CHT oligosaccharides	Passiflora seedlings were sprayed with ~0.007% CHT solution daily 3 times and then inoculated with CMV	CMV virulence halved in plants treated with COS, and the CMV RNA level reduced both in the laboratory and field experiments	[58]
*Chenopodium quinoa* (quinoa)	TNV, CMV	CHT from the Antarctic krill	Plant treatment with 0.1% CHT solution 1 day before inoculation	Reduction in the number of TNV-induced local lesions by 25–50% and CMV-induced lesions by 50–75%	[36]
CHT-based nanoparticles (NPs)					
*Nicotiana benthamiana*	Potato virus Y (PVY)	CHT quaternary ammonium salt NP	Plant treatment with CQAS NPs via root soaking, foliar spraying, or infiltration	More than a 25-fold decrease in viral CP RNA accumulation in plants pre-treated with CQAS NPs,; however, Western blot analysis of CP level demonstrates only a moderate reduction in CP accumulation	[59]
*Nicotiana glutinosa*	AMV	CHT-dextran-NPs were made of 100–300 kDa CHT and dextran sulfate using the ionic gelation method. Hydrodynamic diameter range of the CHT-dextran-NPs was between 20 and 160 nm, with an average diameter of 91.68 nm	Protective (before inoculation), curative (after inoculation), or inactivating (mixed with inoculum) treatment of plants with 100 µg/mL of CHT-dextran NPs	Protective and inactivating treatment gave better results than curative treatment; virus accumulation was assessed via ELISA in extracts from systemic leaves 22 days after inoculation	[60]
*Capsicum annum* (chili pepper)	AMV	CHT-based NPs were prepared from 50 to 190 kDa CHT (75–85% DD). CHT was cross-linked with TPP; CHT-NPs were spherical, with a hydrodynamic diameter of 37.8 nm and a zeta potential of +48.4 mV. CHT-silver NPs (CHT-Ag-NPs) were obtained via chitosan reduction of silver nitrate and were spherical, with a hydrodynamic diameter of 12.55 nm and a zeta potential of +65.1 mV.	Pepper seedlings were treated with 0.1–0.4 mg/mL CHT NPs or 0.05–0.2 mg/mL CHT-Ag NPs 1 day before or 1 day after AMV inoculation or immediately after inoculation	The most prominent effect (90% of inhibition) of CHT-NPs and CHT-Ag-NPs was obtained when they were applied 1 day after virus inoculation (curative effect). Virus accumulation was assessed 21 days after inoculation in extracts of systemic leaves via ELISA	[61]

NS, the type and characteristics of chitosan are not specified; *, CHT did not induce resistance towards tested viruses in *Brassica campestris.*

### 2.2. Dependency of CHT Effect on Its Molecular Weight, Deacetylation Degree, and Charge

CHT’s biological effect strongly depends on its physico-chemical properties: molecular weight (MW), deacetylation degree (DD), and charge [12,62]. Moreover, time, concentration, and mode of plant treatment also make a significant impact on the manifestation of CHT’s antiviral activity.

Chirkov and colleagues reported the positive correlation between the average degree of polymerization and antiviral activity of CHT oligomers (in a range between 15 and 250 units): the longer the CHT oligomer, the higher the antiviral effect [37]. These authors also compared the effect of CHT with an MW of 3, 36, and 120 kDa toward PVX infection and reported that a 120 kDa CHT preparation showed the highest antiviral activity [55]. However, in contrast to these results, Kulikov et al. [38] and Davydova et al. [15] demonstrated that a low-MW fraction of CHT had the most prominent inhibiting effect on viral infection compared to non-fractionated preparation and fractions with higher MW. Such contradiction could have several explanations. First, the difference in the homogeneity of the preparation tested: Chirkov et al. [37,55] characterized samples by the average oligomer length or average MW using viscosimetry, thus, the preparations could contain molecules of different size, while in the later studies [15,38], CHT was fractionated by MW using gel-filtration; thus, the preparation contained only the molecules of the particular MW. Therefore, the homogeneity of CHT preparation plays a significant role in the manifestation of its activity. Second, CHT hydrolysis in all these studies was performed differently. The method of CHT depolymerization affects the CHT preparation’s activity: the products of chemical hydrolysis (using hydrogen oxide) are more bioactive than low-MW CHT obtained via enzymatic digestion (using lysozyme) that could be a result of the difference in their acetylation pattern [15]. In addition, Kulikov et al. [38] showed that CHT monomers glucosamine and N-acetylglucosamine exhibited no antiviral activity. And finally, it should not be completely excluded that the nature of the host–virus interaction may also contribute to the outcome of the antiviral effect of CHT. Indeed, in all three above-mentioned studies, different pathosystems were used: AMV (*Bromoviridae*)/*Phaseolus vulgaris* [37] or BMMV (*Tombusviridae*)/*P. vulgaris* [38] or TMV (*Tobamoviridae*)/*Nicotiana tabacum* [15] which displayed different infection types—BMMV induced systemic infection while AMV and TMV induced local lesions.

However, additional studies on the assessment of the MW–efficiency correlation should be performed.

Another important feature is the deacetylation degree (DD). This property of CHT was demonstrated to be important for CHT’s effect on bacterial and fungal pathogens [63,64]; however, the data on its effect on antiviral resistance induction is still understudied. Chirkov et al. [55] discusses the significance of the DD and suggests that this parameter could be important for CHT’s effect on some plant species (for example, potato) but not on others (bean). This assumption is based on the experiments where the antiviral effect of CHT with DDs in a range from 60 to 98% on bean plants was the same and did not depend on the DD or the CHT source (crab or krill), while for potato plants, CHT activity was different for preparations with a DD of 69% and 85%. However, the later CHT variants also had different MWs, which, as discussed above, may be a more important feature for the induction of an antiviral effect than the DD. Thus, it is likely that the DD is not crucial for the activation of the plant defense response towards viruses unless it is so low that it leads to the abolishment of the CHT molecule’s positive charge. Moreover, Davydova et al. [15] tested CHT preparations of different MWs with various DDs from 98.5% to 75% and observed no correlation between the DD and CHT antiviral activity in contrast to a strong dependence of the CHT effect on its MW, as mentioned above.

Besides MW and DD, the CHT molecule’s charge plays an important role in its antiviral activity as was demonstrated by Chirkov et al. [37]. The effect of anionic and amphoteric CHT derivatives was 100- and 10-times lower compared to cationic CHT of the same MW, respectively. At the same time, a desaminated variant appeared to have the most prominent activity as an inducer of plant antiviral resistance.

### 2.3. CHT-Based Nanoparticles to Fight Plant Virus Infection

Nanomaterials gain more attention nowadays as they enable revealing new properties of well-known compounds. CHT-based nanoparticles (CHT-NPs) could be obtained under specific physical conditions. They are formed when the aqueous CHT solution is mixed with diluted acid and some additional components [19,65,66]. One of the most common methods for CHT-NP generation is ionic gelation [67] which is based on the electrostatic interaction between the amine groups of chitosan and a negatively charged group of a polyanion. Numerous studies on CHT-NPs reported their ability to induce a plant immune response against various pathogens [20,68,69,70]. Moreover, there is evidence of an improved inhibitory effect of CHT-based NPs against fungal pathogens compared with the regular form of CHT [71,72]. However, limited data are present to date on CHT-NPs’ effect against viral infection.

Abdelkhalek et al. [60] obtained composite CHT-dextran-NPs made of 100–300 kDa CHT using the ionic gelation method. *Nicotiana glutinosa* plants were treated with 0.1 mg/mL CHT-dextran-NPs 1 day before, 1 day after, or at the time of inoculation with AMV followed by assessment of symptom severity and virus accumulation levels. It was demonstrated that viral infection was suppressed by 61–100% depending on the treatment protocol. The best results (100% resistance) were shown when CHT-dextran-NPs were applied 1 day before inoculation.

Another study reports that CHT-NPs and composite NPs containing silver (CHT-Ag-NPs) possess high antiviral activity as was demonstrated on chili pepper plants inoculated with AMV [61]. CHT-NPs were obtained via the ionic gelation method from 50–190 kDa CHT (DD 75–85%) using trisodium polyphosphate (TPP). Composite NPs containing silver (CHT-Ag-NPs) were generated from the same 50–190 kDa CHT via reduction of silver nitrate. In contrast to the results of Abdelkhalek et al. [60], the most prominent effect (90% of inhibition as confirmed via ELISA) of CHT-NPs and CHT-Ag-NPs was obtained when they were applied 1 day after virus inoculation. Of note, the difference in the virus inhibition ratio for the assessed CHT-NPs concentrations (from 0.1 to 0.4 mg/mL) was not statistically significant for all groups. This could be explained by the fact that CHT-NPs per se launch the systemic plant response which does not depend on concentration in the tested interval.

Xu et al. [59] generated NPs from CHT quaternary ammonium salt (CQAS-NPs). The antiviral activity of CQAS-NPs was assessed in *N. benthamiana* plants treated with CQAS-NPs via root soaking, foliar spraying, or infiltration and challenged with potato virus Y (PVY). More than a 25-fold decrease in viral RNA accumulation was revealed in the systemic leaves of plants pre-treated with CQAS-NPs. However, these results were not fully supported with Western blot analysis of the PVY coat protein (CP) level: in contrast to RNA, only a moderated reduction in the CP amount was demonstrated. The assessment of CQAS-NPs’ effect was not the main aim of the study thus the data presented are not sufficient for creating the full picture of CQAS-NPs’ activity against viral infection. Thus, future experiments including comparison of the effect of raw CHT and NPs obtained from it are to be performed.

Nevertheless, based on the results described in the abovementioned studies, it could be concluded that NPs made of heterogeneous CHT of high MW (50–300 kDa) with various DDs activate plant resistance towards viruses and other pathogens. CHT-NPs are able to induce plant defense reactions when applied in several-fold lower concentrations than CHT. For example, treatment with a 0.001% CHT-NPs solution (NPs with a Z-average value of 287.3 nm (95.07 nm–307.6 nm)) resulted in the induction of defense related genes, callose deposition, and an increase in total phenol and flavonoid content in *Capsicum annuum* [73]. Moreover, Chandra et al. [68] revealed that a similar or superior biological effect could be reached with a 10-fold lower concentration of CHT-NPs (spherical NPs with a hydrodynamic diameter from 40 to 180 nm with an average diameter of 90 nm) compared to 220 kDa CHT. Thus, NPs are likely to be more effective compared to regular CHT preparations with a similar MW and DD.

### 2.4. Protective, Inactivating, and Curative Activity of CHT

To get closer to unveiling the mechanism of CHT antiviral activity, it is worth taking a look at experimental systems in which these results were obtained (Table 1). In most of the studies, the standard CHT application protocol implies plants’ pre-treatment with 0.1% CHT solution mainly as a foliar spray followed by virus inoculation 24 h later. This approach could be designated as a protective treatment. The second most frequently reported protocol involves simultaneous application of the virus inoculum and a CHT solution as a mixture and is regarded as an inactivating treatment. The last variant—curative—implies spraying of the inoculated plants with CHT solution or their cultivation in the CHT-supplemented medium. The best antiviral effect was achieved when CHT was used as a protective agent (e.g., [36,37,55]) while curative treatment gave no positive results neither with high- nor with low-MW CHT. Only in the case of various CHT-based NPs the curative treatment resulted in a significant effect: a CHT-dextran-NPs foliar application led to a ~60% reduction in virus accumulation [60], while plant treatment with CHT-NPs or CHT-Ag-NPs after inoculation was reported to result in a ~90% inhibition of infection [61]. The “intermediate” variant—CHT addition to the inoculum or inoculation just after CHT treatment—was also rather successful as in the case of CHT [15,42,44] or CHT-based NPs [60,61]. It is reported that an inactivating treatment resulted in a superior inhibiting effect towards viral infection compared to protective treatment [36,45,60,61]. Nagorskaya et al. [45] observed an association of CHT molecules with TMV particles; moreover, along with ordinary virus particles, the authors found virions with morphological abnormalities (swollen or thin virions) in the extracts from inoculated plants treated with CHT. It was suggested that CHT-induced changes in virion morphology could be one of the reasons for the lower efficiency of infection. Unfortunately, no results on virus–NP interactions are available.

## 3. Mechanisms Underlying CHT Biological Effects

Several hypotheses on the CHT antiviral effect are being discussed; however, the exact mechanism is still to be elucidated. While the direct antifungal and bactericidal activity of CHT is demonstrated in numerous studies (reviewed in [62,74,75]) no specific mechanism underlying its antiviral effect is discovered. Thus, presumably, CHT-induced plant resistance towards viral infection is a result of a non-specific plant defense reaction, including systemic acquired resistance, expression of the genes controlling oxidative stress and PR-encoding genes, etc., rather than some direct effect against viruses [25,76]. However, the latter could not be excluded as an “inactivating” protocol of CHT application was reported by several researchers to result in a more pronounced reduction in virus accumulation compared to other treatment protocols as was mentioned above [36,45,60,61]. Moreover, the production of abnormal virions in plants treated with CHT was also registered [45].

### 3.1. CHT Perception and Early Events

CHT is regarded as a general elicitor, and as a pathogen-associated molecular pattern (PAMP) it is perceived via pattern recognition receptor(s) (PRR). At present, the mechanism for chitooligosaccharide sensing was revealed for *A. thaliana*: Lys motif (LysM)-containing receptor-like kinases—AtLYK4 and AtLYK5—form a tripartite complex with a chitin elicitor receptor-like kinase (AtCERK1/LYK1) upon binding of a chitooligosaccharide [77,78,79,80]. A similar model was suggested for rice [81,82]. LysM-RLKs recognizing chitooligosaccharides were isolated from other plants as well [83,84]. In addition, a CHT-binding protein was also identified [49], and it is suggested to be another putative CHT receptor.

The first changes in response to CHT were documented in a few minutes after it was added to the cultured plant cells [85,86]. They include membrane depolarization, modulation of Ca^2+^ and other ions’ balance, a decrease in cytosolic pH, and inhibition of plasma membrane proton pump ATPase activity. Another early event is a CHT-induced increase in the production of reactive oxygen species (ROS)—markers of the first stages of the plant cell response to pathogen attack. ROS accumulation is followed by upregulation of antioxidant genes’ expression and synthesis of ROS scavengers. Both CHT and CHT-based NPs were demonstrated to activate a nitric oxide-dependent defense pathway [48,58,68,73,87]. ROS, Ca^2+^, and nitric oxide are the key players in the induction of programmed cell death (PCD) and, in particular, a hypersensitive response (HR). Characteristic features of PCD—ROS generation and chromatin condensation—were observed in cultured cells, epidermis, or plants treated with CHT [43,85,88,89]. The appearance of PCD features was associated with induced resistance against viruses [43]. Along with the local acquired resistance, the systemic acquired resistance (SAR) develops which results in the upregulation of PR genes as was demonstrated in numerous studies (e.g., [46,47,60,68,70]).

### 3.2. CHT-Induced Activation of Hormone-Dependent Defense Reactions

Salicylic acid (SA) plays a key role in SAR activation [90,91]. There are two main pathways of SA biosynthesis; one is mediated by phenylalanine ammonium lyase (PAL) and the other by isochorismate synthase. The ratio of SA synthesized via each pathway varies between species [92]. CHT was shown to activate PAL expression [46,47,60,93]. CHT oligomers induce resistance to TMV in wild-type *A. thaliana* plants but fail to affect *NahG Arabidopsis* which is regarded as a model deficient in the salicylate pathway [48]. In addition, in the same study, it was shown that the *Arabidopsis* mutant *jar1* lacking the jasmonic acid (JA) pathway responds to CHT treatment similar to the wild-type plants indicating that it is the SA pathway but not the JA pathway that is important for CHT-dependent induction of resistance to TMV. In contrast to these results, several studies reported activation of the octadecanoic pathway leading to increased JA content in plant tissues after CHT application [94,95,96]. Finally, CHT oligosaccharides were demonstrated to induce both the JA and SA pathways in *A. thaliana* [97]. Despite SA and JA being regarded as antagonists in many plant reactions to stress, the fine-tuned defense signaling sometimes implies their synergy and some kind of trade-off between them depending on the attacking pathogen [90].

In addition to SA and JA signaling activation, it was shown that CHT affects other hormones’ pathways. Abscisic acid (ABA) plays a significant role in plant reactions in response to abiotic and biotic stress, in particular, viral infection [98]. Thus, special attention to ABA should be paid in the context of CHT antiviral activity as well as interconnection between ABA and callose (the latter is discussed below). An increase in ABA content after CHT application was reported by Iriti and Faoro [39,76], and it correlated with plant resistance to TNV. Kuyyogsuy et al. [93] revealed ABA accumulation in *Hevea brasiliensis* tissues in response to CHT.

Genes whose expression affects indolile acetic acid and giberillic acid synthesis were identified among those responsive to CHT treatment [64]. Zhang et al. [58] analyzed the transcriptome and proteome of passiflora and showed that CHT oligosaccharides activated brassinosteroids (BRs) cell signaling pathway. BRs are perceived via BR1 receptor protein kinase on the plasma membrane which further leads to (1) the enhanced production of ROS; (2) upregulation of mitogen-activated protein kinase (MAPK); and (3) stimulation of nitric oxide (NO) generation and enhancement in the antioxidative defense system [99]. Thus, BRs pathway activation results in the enhanced local production of H_2_O_2_ and NO followed by development of systemic reactions due to the transfer of these signal molecules to the upper leaves of the plant inducing antiviral defense [100]. The data on ethylene involvement in CHT-induced reactions seem to be controversial: Iriti et al. [101], using chemical inhibitors, showed that the ethylene pathway is not involved in CHT-induced resistance to a virus, while in a more recent study, Czékus et al. [102] demonstrated that CHT treatment launched ethylene emission and suggested an important role of ethylene in the establishment of local and systemic acquired resistance in response to CHT.

Thus, there is a complicated interplay between CHT-mediated activation of hormone crosstalk leading to plant protection against various pathogens and resistance to multiple abiotic stresses.

### 3.3. The Role of Callose in CHT-Mediated Plant Defense

Numerous studies report that CHT induces callose synthesis [37,39,103,104]. It was shown for the bean/TNV pathosystem that CHT application leads to the accumulation of ABA in plant tissues, downregulation of the callose-degrading enzymes, 1,3-β-glucanases, activity, and enhanced callose deposition that correlates with resistance to TNV [39]. In *A. thaliana*, ABA was demonstrated to suppress *1,3-β-glucanase* gene expression and promote callose accumulation [105]. Callose is a well-known regulator of plasmodesmata (PD) permeability: an increase in PD callose deposition leads to the reduction in intercellular transport of macromolecules, therefore, restricting viral local spread [106]. Correlations were observed for virus accumulation, callose depositions, and 1,3-β-glucanase activity in leaves treated with CHT: reduction in PVX content was associated with increased callose in the background of decreased β-1,3-glucanase activity. Noteworthy, 1,3-β-glucanase and callose were assessed just after virus inoculation (in 15 min), while PVX content was determined 6 days after inoculation. Thus, the level of β-1,3-glucanases had been changed at the moment of virus inoculation, “preparing a background” for the infection and, therefore, affecting infection development [55]. However, callose-dependent downregulation of viral movement is definitely not the only mechanism underlying the CHT-induced effect because it was demonstrated that CHT treatment of the protoplasts which lacks a cell wall, and thus PD, nevertheless resulted in their partial resistance to TMV infection [42]. Notably, an ABA inhibitor applied before CHT reduced but did not completely cancel CHT antiviral effect [39], indicating that there are other defense pathways launched by CHT.

In contrast, other studies on CHT and CHT-NPs effects report upregulation of callose-degrading 1,3-β-glucanase activity [107,108,109,110]. The authors discuss 1,3-β-glucanases as PR-2 proteins playing in the orchestra of the plant defense against fungal and bacterial pathogens. The contradictory data on 1,3-β-glucanase levels might arise as a result of differences in the experimental conditions and properties of the CHT used. For example, Faoro and Iriti [41] showed that low-MW (6 and 22 kDa) CHT did not induce callose deposition, while CHT of 76, 120, 139 kDa did. Moreover, 1,3-β-glucanase activity and callose content vary significantly depending on the time passed from CHT treatment [55]. Chandra et al. [68] showed that 1,3-β-glucanase enzymatic activity increased by ~29% and 34% in 24 h after treatment with either CHT (0.01%) or CHT-NPs (0.001%), respectively. And finally, Jogaiah et al. [107] reported that both callose content and 1,3-β-glucanase enzymatic activity increased in response to CHT treatment.

Noteworthy, the family of 1,3-β-glucanases comprises several dozen members that share structural similarity but a high diversity of temporal and tissue-specific expression patterns. This diversity indicates that 1,3-β-glucanases respond to different stimuli and have various biological roles [111]. A large number of 1,3-β-glucanases fall into the group of PR-2 proteins, while others participate in PD callose hydrolysis and regulation of PD permeability. Thus, some 1,3-β-glucanases could be activated while others are downregulated in response to the same stress factor or environmental stimuli. This could partially explain the controversy in the results, reported by different authors, as mainly it was enzymatic activity in the plant extracts that was assessed and that reflected the total sum of either up- or downregulated 1,3-β-glucanases possessing various functions and intracellular localization.

### 3.4. Putative Mechanisms of CHT Antiviral Activity

Thus, multiple pathways are activated after CHT application, most of them are connected with plant innate immunity activation. Moreover, the plant response to CHT markedly varies between species. Therefore, it is hard to distinguish which of these processes in particular underlie the acquired resistance against viruses. As mentioned above, CHT induces accumulation of SA and activates SAR. However, SAR significance in the antiviral plant response varies depending on the host–virus combination and the type of infection: SAR appeared to be more effective in virus–host systems characterized with localized infection (HR) than in case of systemic infection [24,112]. At the same time, SA was demonstrated to play an important role in antiviral defense being a signal molecule that activates multiple pathways and initiates various changes in plant cell physiological status and gene expression [92]. SA was reported to negatively affect replication, viral intercellular spread, and systemic movement [113,114,115,116]. Notably, the particular stage of viral infection that is affected by SA depends on the virus–host combination. Thus, it could be hypothesized that the different extent of CHT activity towards various virus–host pairs may be partly explained by this feature of SA-mediated antiviral defense. Furthermore, viral infection per se activates host defense responses that could involve the same pathways as CHT does or induce the finely tuned interplay between host defense and virus counter-defense.

**Figure 2:**
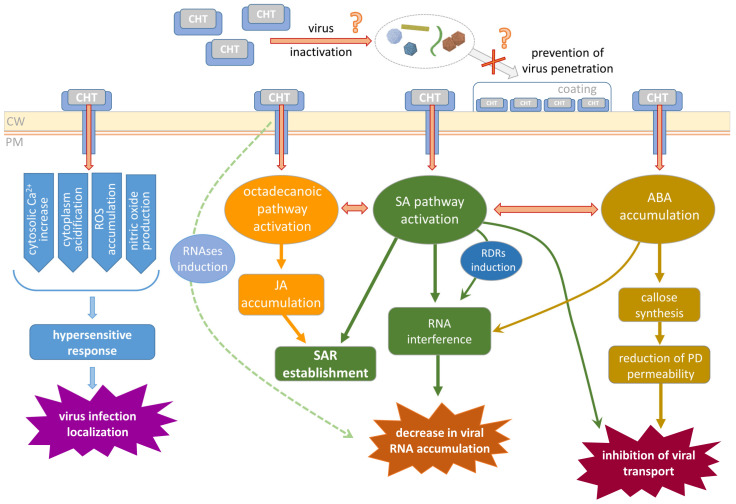
Schematic representation of the putative mechanisms related to CHT-mediated plant resistance to viral infection. CW, cell wall; PM, plasma membrane; ROS, reactive oxygen species; RNAses, ribonucleases; SA, salicylic acid; JA, jasmonic acid; ABA, abscisic acid; RDRs, RNA-dependent RNA polymerases; SAR, systemic acquired resistance; PD, plasmodesmata.

From a bird’s eye view, viral infection contains three major stages: cell penetration, reproduction, and transport. CHT could interfere with any of these processes (Figure 2). First, it potentially could prevent cellular penetration as it was shown that CHT forms a film on the leaf surface when applied as a spray. However, in most of the experiments, virus inoculation is performed using some abrasive to wound the leaf and damage the surface (cuticle and cell wall) guarantying successful penetration of the virions. It is possible that the CHT layer would partially protect the leaf from virus entry mechanically (if the abrasive is not used) or would bind virions somehow affecting their viability.

Second, CHT-induced reactions interfere with virus performance inside the cell. Virus reproduction includes multiple events; its efficiency depends on viral RNA replication and stability, availability of the essential cellular factors, and intensity of the cellular defense responses (RNA interference, for example). CHT was demonstrated to induce synthesis of a putative cellular RNA-dependent RNA polymerase (RDR) [42], the enzyme participating in one of the major mechanisms of antiviral defense—RNA interference leading to degradation of the viral RNA. On the other hand, SA plant treatment also induces RDR1 and RDR6 in some species [117,118,119]; thus, it could be speculated that CHT-mediated activation of RDR in protoplasts [42] may be mediated by SA. In addition to SA-mediated inhibition of virus replication and priming of silencing [92], recent studies have linked another hormone, ABA, to the antiviral silencing pathway, which interferes with virus accumulation, and the micro RNA (miRNA) pathway through which ABA affects the maturation and stability of miRNAs [98]. CHT treatment also was shown to result in the increased activity of ribonucleases that could negatively affect viral RNA accumulation [55].

Finally, a stage that is very important for efficient propagation is viral movement within the plant. It includes intercellular local spread and systemic transport and could also be a target of CHT action. As was mentioned above, CHT induces callose synthesis [37,39,103,104] and its deposition around PD, which leads to a decrease in PD permeability, thus preventing viral cell-to-cell transport. Moreover, SA was reported to induce the restriction of TMV infection to the primary-infected mesophyll cells independently of PD regulation or interference with movement protein function [114]. Another mechanism of CHT indirect influence on virus local spread could be suggested based on the study of oligochitosan-induced protein kinase (OIPK) isolated from tobacco [120]. OIPK was shown to play an important role in plant resistance to TMV as its upregulation led to less effective TMV infection [121]. It could be speculated that OIPK being a Ser/Thr kinase participates in TMV movement protein phosphorylation regulating its ability to perform PD gating and mediating TMV local spread.

## 4. Conclusions and Perspectives

In context of plant–pathogen interactions, CHT and its derivatives are regarded as an attractive tool for activation of different branches of the plant immune response. Moreover, CHT showed high efficiency as an inductor of plant resistance to viral infection. Most of the tested pathosystems that include diverse viruses and host plants are sensitive to CHT albeit to a different extent that depends on the properties of CHT preparation and the protocol of application. It is believed that a general non-specific plant defense response underlies CHT antiviral activity; however, the exact mechanisms and pathways responsible for it are still far from clear. CHT is an elicitor that launches a network of phytohormone crosstalk. Numerous studies report activation of defense-related genes and enzymes involved in hormones’ biosynthesis and hormone-mediated pathways. Therefore, it is hardly possible to distinguish between multiple consequences of CHT perception by the plant cell and to define which branch in particular is important for CHT-induced resistance to viral infection. In addition, viruses induce diverse defense reactions in plants, including accumulation of such signaling molecules as SA, JA, and ABA, activation of HR, or establishment of systemic infection. Therefore, these responses could interfere with or enhance CHT-mediated effects, resulting in complication of the mechanism underlying the interaction between the plant and virus in CHT-treated plants. Moreover, some results on CHT effects obtained at the moment are inconsistent with each other and even controversial. One of the main difficulties that does not allow analyzing and comparing the bulk of data obtained by different research groups is the difference in experimental set-up, protocols, and, finally, the physico-chemical properties of CHT preparations used in the studies. For example, it is generally accepted that CHT polymerization degree and MW are crucial for its efficiency as an antiviral agent, but it is still debated what variant is more potent. A significant advance made in the field of CHT application is the generation of CHT-based nanomaterials. This approach is believed to be advantageous because CHT-NPs are likely to show better performance compared to the “raw” CHT. However, there are only a few studies in which the direct comparison of CHT-NPs and CHT antiviral activity is performed; thus, more research on this subject is needed.

Although CHT is gaining more and more attention as a basis for environment-friendly pesticides, herbicides, fertilizers, and a platform for delivery of various cargo, a lot is to be studied before it becomes a commonly used compound. More research is to be performed to prove CHT and its derivatives are really safe for the biosphere, to assess their effect on the environment, and to comprehend how CHT is “processed” once being applied in the field. Another hurdle for the transfer of CHT-based formulations from the laboratory to the field is the cost of its large-scale production: despite the main “precursor” of CHT—chitin—being widely available, its downstream processing to obtain the homogenous CHT preparation with desirable properties is rather cost-consuming. Search for novel CHT sources and development of the technologies that allow for the reduction in CHT production costs and to increase the volume are the tasks of high significance on the way to wide application of this polymer. On the other hand, CHT derivatives and CHT-based nanomaterials might solve this problem as (1) the biological activity of CHT-NPs is likely to be higher; (2) CHT could be a basis for obtaining more potent derivatives; (3) the range of the CHT MWs for generation of a homogenous preparation of CHT-NPs could be rather wide; and (4) composite CHT-based NPs supplemented with metals or other polymers with novel properties and higher efficiency could be produced.

Thus, CHT could be and should be regarded as a prospective ingredient for the development of bioactive formulations aimed to protect agricultural plants from various pathogens and especially from viral infection. In addition, treatment of seeds or leaves with CHT-based preparations would lead to other beneficial effects such as an increase in biomass and the content of active components (polyphenols, antioxidants, etc.) in cultured plants. As CHT is believed to be biodegradable, biosafe, and biocompatible, even partial substitution of traditional chemical pesticides with CHT-based products promises to be a step towards sustainable agriculture.

## Data Availability

Not applicable.
